# Analysis of light emission and Schlieren from short gap high voltage streamers representing lightning impulses

**DOI:** 10.1038/s41598-021-03839-y

**Published:** 2021-12-21

**Authors:** D. Mitchard, P. Widger, A. Haddad

**Affiliations:** Advanced High Voltage Engineering Research Centre, School of Engineering, Cardiff University, Wales, CF24 3AA UK

**Keywords:** Electrical and electronic engineering, Characterization and analytical techniques, Experimental particle physics

## Abstract

Light emissions and Schlieren structures were simultaneously observed from streamers produced by tens of kilovolts 1.2/50 μs impulses, representing the high voltage component of lightning, applied across a 4 cm air gap between a variety of electrode geometries and a ground plane in an unconfined environment. The results demonstrated that the light emissions and Schlieren structures coincide along the same streamer filaments but on different timescales; the light existing only during the microsecond timeframe impulse whereas the Schlieren continued to develop into the millisecond timeframe, moving towards the centre of the air gap whilst diffusing into the surrounding air within 100 ms. If an electrical breakdown did occur, the Schlieren structures outside the arc remained visible. Streamer formation theory for high voltage impulses is subsequently refined to include the observed Schlieren mechanism.

## Introduction

Lightning is one of the most powerful and destructive naturally occurring electrical phenomena, and the protection of vital infrastructure, such as power transmission and transportation, from strikes and destructive effects is of great importance. However, given its unpredictability, it can be very demanding to carry out detailed studies of the underlying mechanisms and characteristics of lightning and related phenomena in the natural environment. Laboratories have been developed to study the high current and high voltage components individually, which are often used to represent the physically destructive aspects through joule heating, electromagnetic forces, and electric field breakdown effects in both conducting and insulating materials. This is because the production of a combined high current and high voltage arc as seen in a natural lightning strike are beyond the capabilities of any existing laboratory generator. A laboratory high voltage component can consist of impulses up to the megavolt magnitude range, but most frequently tens to hundreds of kilovolts are used which last hundreds of microseconds. These can, for example, be used to understand whether a power substation component may fail by creating a fault-like overvoltage condition. In such a scenario, a lightning strike on one part of a power network can result in partial or full discharge events in other parts of the network, such as gas insulated switchgear. Applying a high voltage induced field to test such components can determine the insulation strength of an insulating gap, such as air or a solid insulation material, and provide statistical evidence as to the likelihood of an electrical breakdown to ground. The mechanism by which the air breakdown would occur is through the formation of transient streamers, lasting for only a few microseconds, within the air gap between the high voltage electrode and ground which, if the conditions are right, could create a conductive channel between the two.

**Figure 1:**
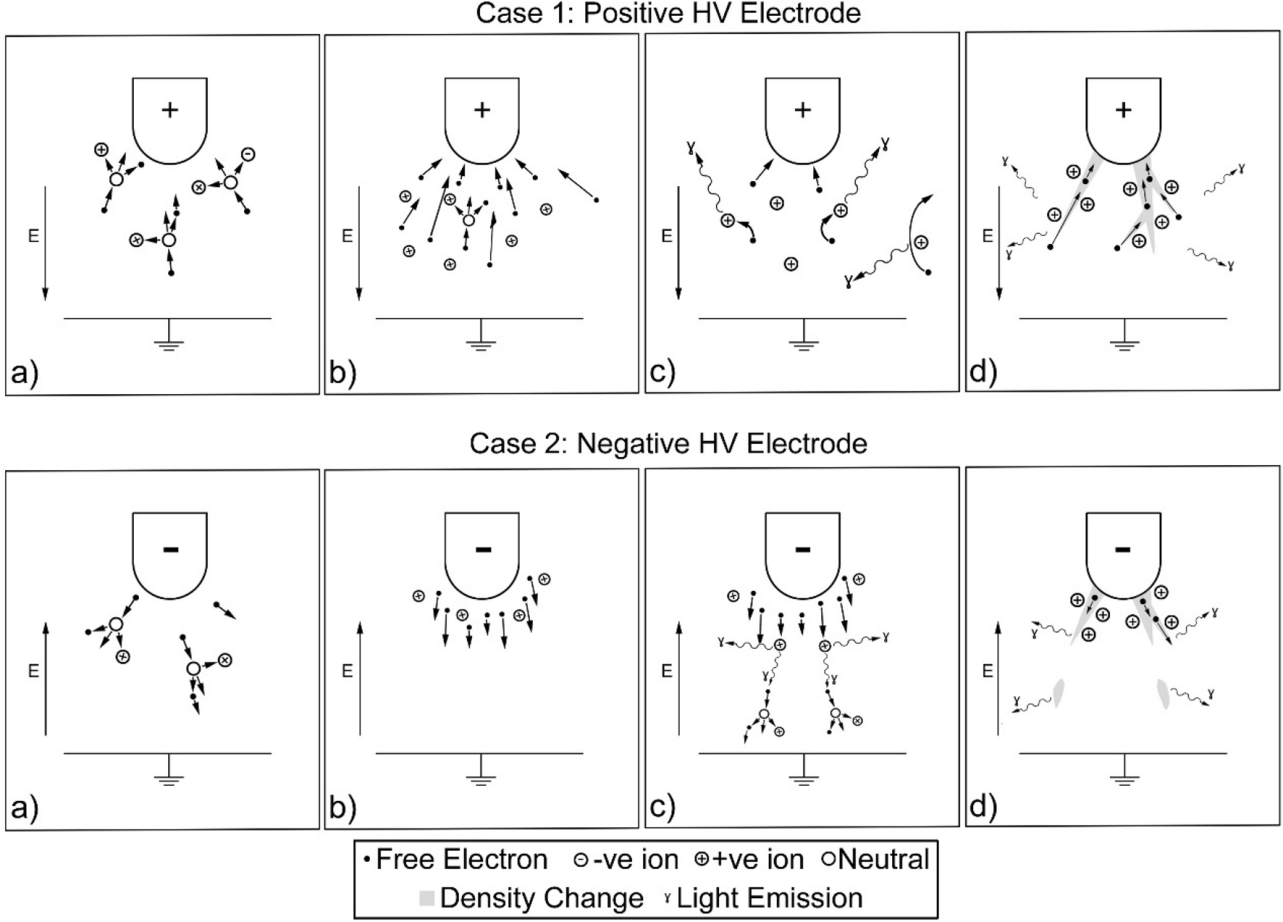
The formation of positive and negative streamers between a high voltage electrode and ground plane across an air gap. (**a**) Free electrons accelerate towards/away from the electrode creating ions, (**b**) electrons accelerate and collide with more molecules creating an avalanche, (**c**) recombination of ions and Bremsstrahlung radiation emitting light and (**d**) formation of filament structures^3^.

Streamer formation^1–3^ in positive and negative electric fields, for example, in an air gap between a high voltage electrode and ground plane, differ due to the mobility of heavier positive ions and lighter electrons, as illustrated in Fig. 1. For positive streamers, free electrons in the air are accelerated towards the positive electrode and collide with neutral molecules liberating more electrons and creating positive ions. The freed electrons, in turn, accelerate and collide with other molecules creating an avalanche called a Townsend discharge^1^, with the large number of positive ions forming an additional electric field which intensifies the ionisation process. Some lower energy electrons may recombine with positive ions emitting photons of light, typically in the ultraviolet to visible wavelength range. Light in the same wavelength range can also be emitted via Bremsstrahlung radiation. Filament structures of charge particles grow outwards from the electrodes and can branch in different directions, including successive avalanches in the regions ahead of the streamer. As the rate of ionisation exceeds the rate of recombination, the current density rises and the air is heated, reducing density, and increasing conductivity of the streamer. In certain scenarios, such as for sufficiently high fields across long gaps, one or more of the filaments may turn into a highly conducting leader which could then result in an electrical breakdown. For negative streamers, a single free electron is accelerated away from the negative electrode colliding with neutral molecules and creating a localised avalanche and additional field. Electrons can also be freed from the surface of the electrode. Further electrons ahead of this region are liberated by photons from the first avalanche creating another localised field. This process continues onwards whilst electrons are accelerated between the regions creating filament structures which grow away from the electrodes. As with positive streamers, the current density rises within the negative streamer reducing its density and increasing its conductivity and, in certain scenarios, a leader may be formed which could result in an electrical breakdown. The intricacies of these mechanisms can be complex, and some examples of recent work in this area are given in^4–6^. Electrode geometry is also known to affect the structure of streamer filaments as this alters the structure of field lines within the air gap. Sharp edges, such as at the point of an electrode, create quasiuniform fields resulting in narrower and more direct streamer structures. Flat or rounded edges, such as around a hemispherical electrode, create highly divergent fields resulting in broader structures.

The observation of both light emissions and Schlieren structure have traditionally been used to study streamers and other related phenomena^7–9^, with recent examples including using optical cameras to image streamers in air^10–12^ and Schlieren imaging to analyse streamer and leader structure^13–16^. They are sometimes used interchangeably to derive characteristics but are rarely used simultaneously. Although both light and Schlieren imaging can be used to observe the structure and shape of streamers, with both being a result of streamer formation, the underlying mechanisms for each are very different. The emitted light is a result of either the recombination of atoms or Bremsstrahlung radiation and is often visible to the naked eye as a blue-white flash which can be captured by a wide range of cameras. Schlieren is an optical effect within the air caused by localised density changes typically from thermal or shock effects and is much harder to see but is sometimes visible by observing light from an external source being refracted at differing angles through the Schlieren. A specialised optical setup using filters, knife edges, mirrors and/or lenses are typically required to capture the effect reliably on camera.

In this paper, a high voltage generator was used to apply both positive and negative high voltage 1.2/50 impulses to an electrode of three different geometries; round, flat and point, positioned 4 cm from an opposing 50 × 50 cm ground plane. The experiments were carried out in an unconfined environment, i.e., outside of a test chamber, to minimise the effect of shockwaves reflected from nearby surfaces interfering with the results. The breakdown voltage, governed by the critical reduced field strength of the air at that voltage, for each polarity and geometry was found experimentally such that the electric field could be applied as close to this value as possible without a breakdown occurring. A 1.2/50 µs waveform conforming to the BS60060-1 standard for high voltage lightning impulses^17^ was used in the approximate range of − 65 to + 50 kV. Further details of this waveform including a spectral analysis can be found in^18^. The voltage waveform was recorded via a capacitive voltage divider with a ratio of 27,931:1 V and resolution in the volts range connected between the high voltage generator and electrode, whereas the current was measured by a current transducer with a ratio of 0.1:1 A and resolution in the milliamps range positioned around the isolated earth lead connecting the ground plane to earth. A pair of synchronised cameras designed to trigger at the same time as the application of the high voltage impulse were used to simultaneously capture the resulting streamers. The cameras captured a series of still images at a rate of 21,000 fps resulting in each image lasting 47.62 µs, with a timing error of 10.0 µs between them. The first camera was positioned to observe directly the visible light emitted from the streamers whereas the second was positioned to observe the Schlieren effect through a specialised optical setup consisting of an LED point light source, two 13 cm diameter biconvex lenses and a knife edge. It is important to note that the images from the Schlieren camera are those of air density differentials, with larger differentials being more prominent. Experiments were carried out to observe both the visible light emissions and Schlieren structures of the resulting streamers. The lens on the second camera was then adjusted and a second set of experiments were carried out to observe the development of the Schlieren structures over a longer period. Schlieren structures were also observed during several electrical breakdowns. All images were enhanced using post-processing image subtraction and brightening techniques.

## Results

### Comparison of light emissions and Schlieren structures

**Figure 2:**
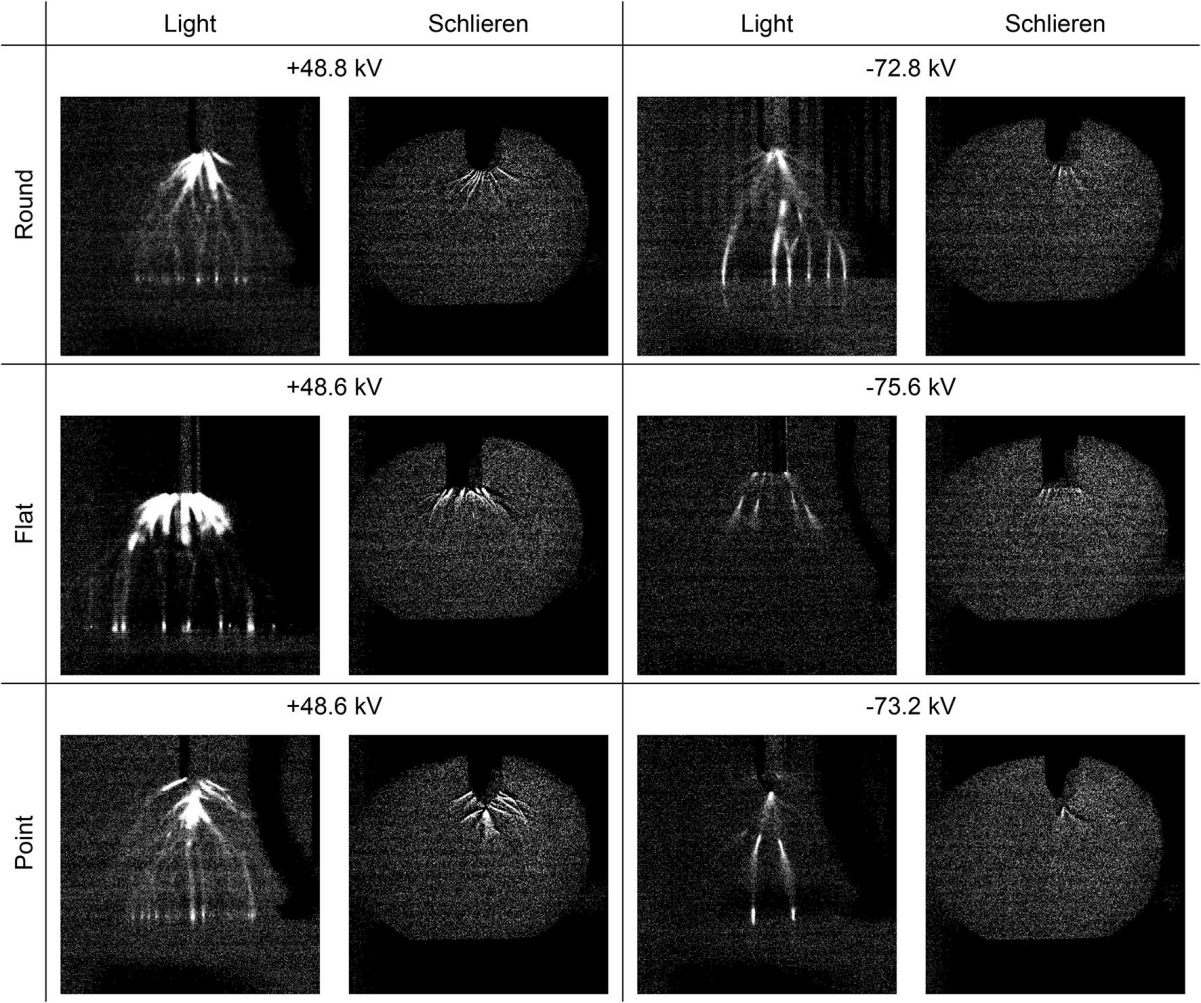
Light emissions and Schlieren structures for positive and negative high voltage impulses applied to round, flat and point electrodes opposite a ground plane. All images are the first frame following the high voltage impulse. The applied peak voltage impulse for each, which were all 0.4 kV below the experimentally determined breakdown voltages, are displayed above each pair of images. The images have been enhanced by image subtraction and 40% brightness increase for clarity.

Both positive and negative high voltage impulses were applied to each electrode geometry. Although the critical reduced field strength of ambient air is typically around 33 kV/cm, it can vary due to polarity, geometry, temperature, humidity, etc., and so it was found experimentally by stepping up the voltage by 0.4 kV and applying an impulse until a breakdown occurred. Once this had happened, the voltage was stepped down by 0.4 kV and a further impulse applied which produced visible streamers, usually without a breakdown occurring. Both cameras were then used to image the light emissions and Schlieren structures simultaneously, and the voltage and current profiles were recorded. In all experiments, the light emissions observed by the first camera and Schlieren structures observed by the second camera were both seen at the same time as the high voltage field was applied. However, the light emissions were only observed during the first frame, i.e., they were present for less than 47.62 µs, whereas the Schlieren structures were observed from the first frame onwards. The first Schlieren frame consisted of both light emissions and Schlieren structures whereas the second Schlieren frame consisted of only the Schlieren structures. As there was no real change in the Schlieren structures in the frames that immediately followed, the first light emission frame, displaying only light emissions, was compared to the second Schlieren frame, displaying only Schlieren structures. Examples of the data obtained are presented in Fig. 2.

**Figure 3:**
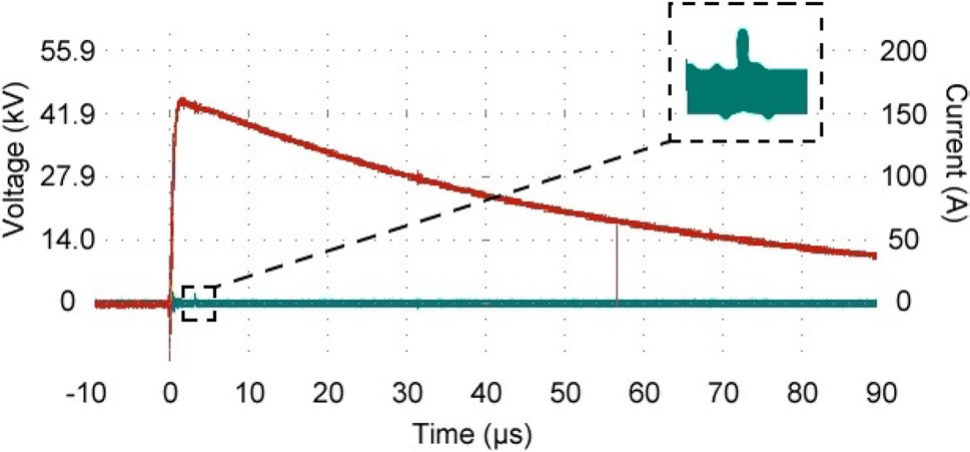
The high voltage impulse profile, conforming to the BS60060-1 electrical standard^17^ (red), and the current profile exhibiting small spikes consistent with a relatively small flow of charge from ionisation of the air (green and zoomed area).

The high voltage impulse profile, as measured by the voltage divider, was verified to conform to the BS60060-1 electrical standard in all cases. The current profile, as measured by the current transformer, exhibited several small spikes in the Ampere range, consistent with a relatively small flow of charge resulting from ionisation of the air, often classified as partial discharge. These partial discharges were recorded from the grounding cable connected to the experimental test object and were measured using an isolated current transformer. The grounding of the test object was directly connected to a star grounding point for the impulse generator which, as in many high voltage laboratories, can result in the partial discharge superimposing itself back onto the high voltage waveform. The partial discharges observed on the current profile could not be perceived as measurement noise because they could be directly attributed to a recorded Schlieren event on the cameras, which was a completely isolated system. An example of these profiles is given in Fig. 3. It can be seen from Fig. 2 that both the light emissions and Schlieren structures coincide along the same streamer filaments as expected. However, there is light emitted from parts of these filaments where no Schlieren effect could be seen, notably in areas further from the high voltage electrodes. This would indicate that, even though filaments have been formed which connect the electrode and ground plane, significant density change within these filaments had only started to occur where the field is most intense, i.e., immediately next to the high voltage electrode. There is also a visible distinction between light emissions for positive and negative streamers in Fig. 2. The positive streamers consist of uniform channels emanating from the high voltage electrode and, in some cases, from the ground plane. These channels decrease in intensity the further away they are from the electrode and plane, whereas the negative streamers consist of distinct areas of more intense light emissions within the air gap. This is consistent with the current theory as illustrated in Fig. 1.

### Analysis of Schlieren structures

**Figure 4:**
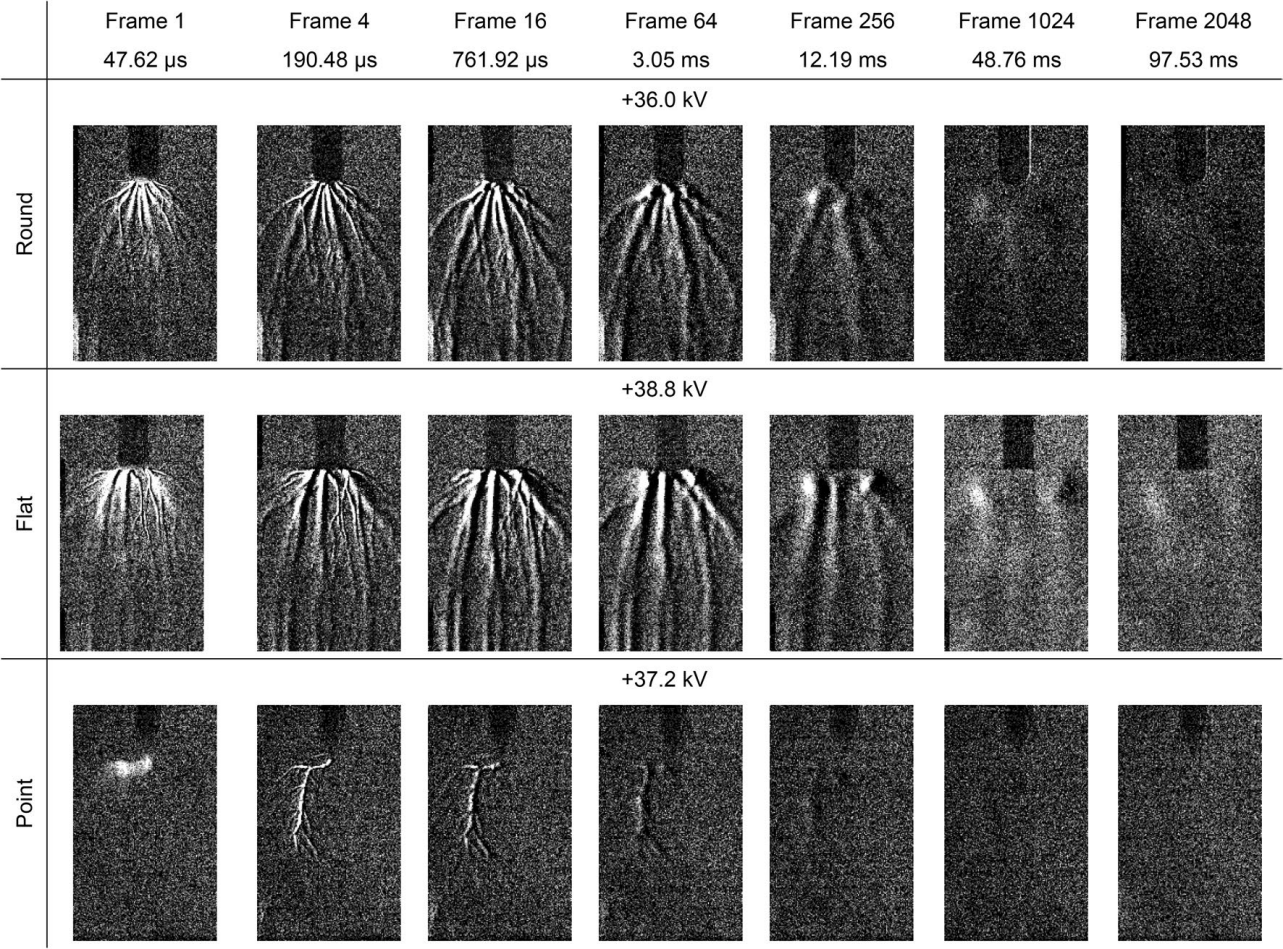
Schlieren structures over time for a positive high voltage impulse applied to round, flat and point electrodes opposite a ground plane. The applied peak voltage impulse for each, which were all 0.4 kV below the experimentally determined breakdown voltages, are displayed above each image. The images have been enhanced by image subtraction and 40% brightness increase for clarity.

**Figure 5:**
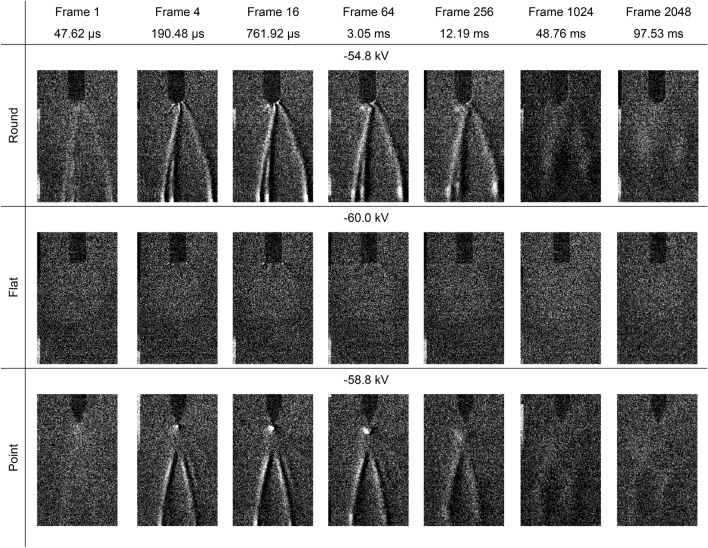
Schlieren structures over time for a negative high voltage impulse applied to round, flat and point electrodes opposite a ground plane. The applied peak voltage impulse for each, which were all 0.4 kV below the experimentally determined breakdown voltages, are displayed above each image. Note that for the flat electrode, Schlieren does exists but is only barely visible near the corners of the electrode. The images have been enhanced by image subtraction and 40% brightness increase for clarity.

The settings on the second camera were adjusted to give a magnified view of the Schlieren structures as well as an extended running time up to one second after the high voltage impulse had been applied, with the first camera no longer being used. The above experiments were repeated using the same high voltage 0.4 kV stepping method, although the point electrode was replaced with a second, sharper point electrode to minimise streamer formation from the sides as seen in Fig. 2. Examples of the data obtained over time for positive high voltage impulses are presented in Fig. 4 and negative high voltage impulses are presented in Fig. 5. Note that, in these figures, the first Schlieren images also include the light emissions.

In all cases, the Schlieren structures were found to continue developing into the millisecond timeframe along filaments which connect between the previously energised electrode and the ground plane. Data from the current transducer showed that there was no charge flow greater than the approximately 0.5. A noise level of the current transducer during this period, indicating that if any charge were flowing then it would be very low. There were notable density changes, which appear as brighter regions, not only along the filaments but also at both the high voltage electrode and ground plane. This is reminiscent of the filaments seen in the light emission images presented in the previous section, despite the light only being emitted in the first frame, i.e., within 47.62 µs, and the high voltage impulse having completely diminished by the fourth frame at 190.43 µs. The Schlieren structures then expand in a perpendicular direction to their alignment, i.e., horizontally, whilst moving towards the centre of the air gap in a parallel direction, i.e., vertically, eventually dissipating into the surrounding air over tens of milliseconds before disappearing completely within 100 ms. In this respect, the Schlieren structures can be considered a longer-lived imprint of the short-lived streamers within the air.

**Figure 6:**
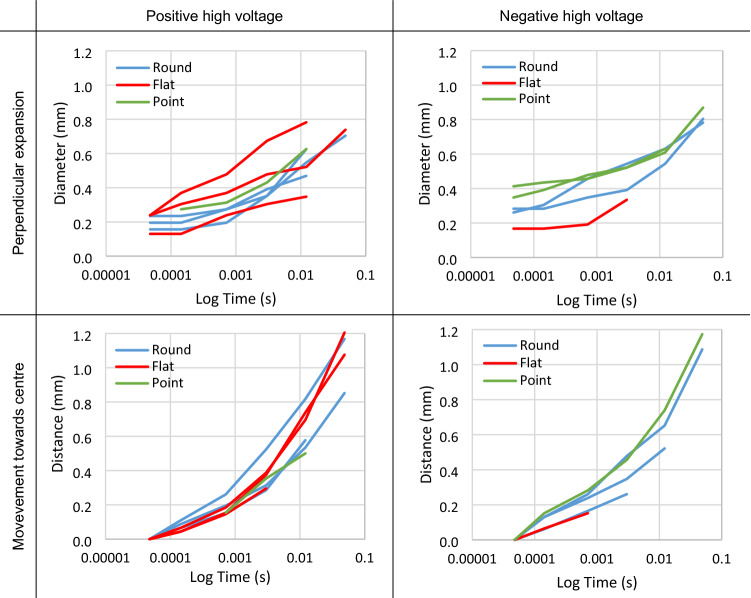
The perpendicular expansion of the filaments and movement of brighter regions towards the centre of the air gap of the Schlieren structures over time for round, flat and point electrodes, as measured from the data presented in Figs. 4 and 5.

The perpendicular expansion of the Schlieren structures can be tracked from one image to the next by measuring the diameter of the more prominent filaments at the same point in each image. Similarly, the movement of brighter regions towards the centre of the air gap can be tracked by measuring how far the centre point of the region has moved away from its starting position in the first image. Plots of both the perpendicular expansion and movement towards the centre are shown in Fig. 6.

### Schlieren structures during electrical breakdowns

**Figure 7:**
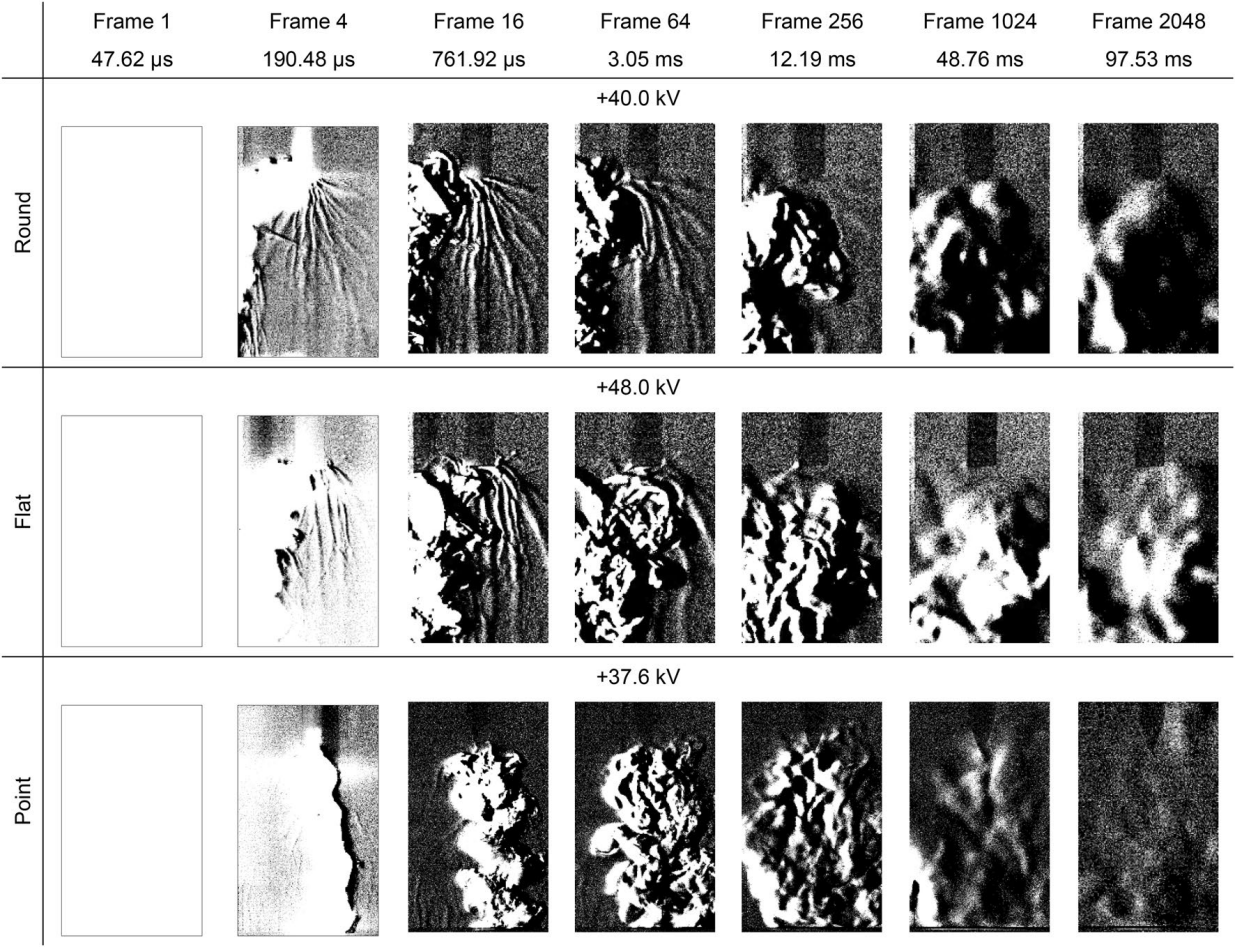
Schlieren structures over time for a positive high voltage impulse applied to round, flat and point electrodes opposite a ground plane when an electrical breakdown has occurred. The first images in each case are saturated. These images have been enhanced by image subtraction and, from frame 16 onwards, a 40% increase in brightness for clarity.

During the experiments, several electrical breakdown events were captured by both cameras. Such events emit a large amount of light within the first frame which saturated both cameras in the first set of experiments. However, during the second set of experiments, the Schlieren camera was able to capture data following the breakdown, as demonstrated in Fig. 7. In most cases, typically when the breakdown occurs to one side of the electrode, the Schlieren structures connecting the electrode to the ground plane are still visible after the high voltage impulse has diminished, as seen in previous experiments when no breakdown occurred. This would indicate that, although many filaments existed, the conditions were right for one to become a current conducting channel, heating the air and increasing the conductivity to a point where a current could flow between the electrode and ground plane. The Schlieren imprint then remains in place until either the turbulent air of the breakdown disrupts it, or it dissipates into the surrounding air.

## Discussion

The results of the experiments described in this paper highlight a more intricate streamer formation process for high voltage impulses across short gaps, such as those representing the high voltage component of lightning, building on existing theory. This does not necessarily apply to other applications where streamers may exist, such as other impulses, alternating current and direct current. It is important to note that the steps described by the existing theory, as shown in Fig. 1, have been observed. The electrode geometry has also affected the streamer formation as expected, with the point electrode producing a smaller number of more direct streamer filaments and the flat and round geometries producing a larger spread of streamer filaments.

**Figure 8:**
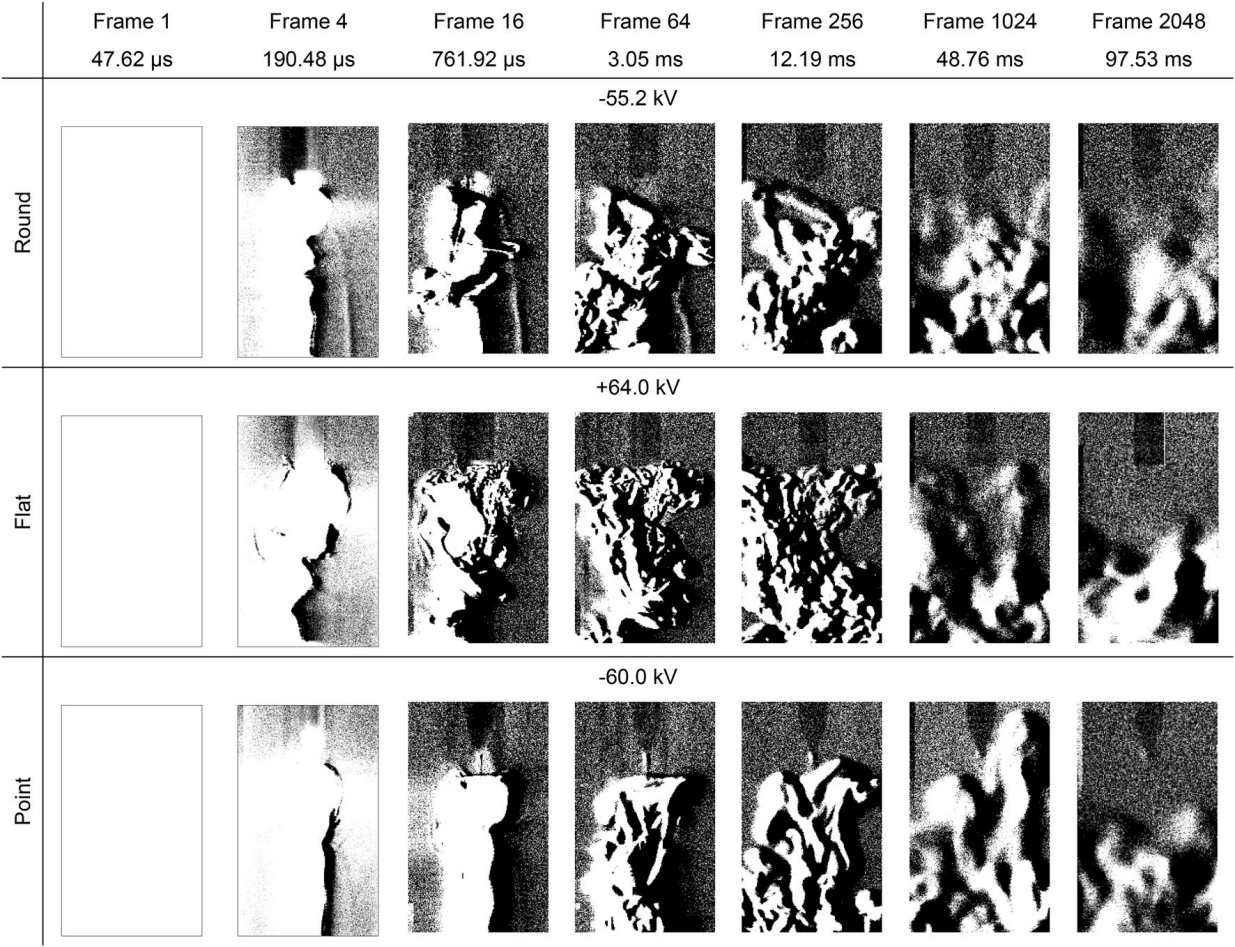
Schlieren structures over time for a negative high voltage impulse applied to round, flat and point electrodes opposite a ground plane when an electrical breakdown has occurred. The first images in each case are saturated. These images have been enhanced by image subtraction and, from frame 16 onwards, a 40% increase in brightness for clarity.

Figure 2 demonstrates that the visible light emissions and the Schlieren structures coincide along the same streamer filaments which branch outwards from the electrodes. In the case of negative streamers, separate distinct areas of more intense light emissions are seen within the air gap. However, the light images show filaments connecting the energised electrode and the ground plane whereas the Schlieren images only show filaments around the high voltage electrode. Comparing the two, the Schlieren structures coincides with the brightest parts of the filaments around the electrode where the field is more intense, indicating that the ionisation process has started to cause localised heating resulting in a change of air density. However, Figs. 4 and 5 demonstrate that, although the ionisation and recombination process has largely diminished due to the rapidly decreasing high electric field in the microsecond timeframe, the Schlieren structures continue to develop along the filaments into the millisecond timeframe. Many of these filaments connect the high voltage electrode and ground plane reminiscent of the light images in Fig. 2, with brighter regions at both the high voltage electrode and ground plane. This mechanism continues even after an electrical breakdown has occurred along other filaments, with the Schlieren structures remaining in place unless disturbed by the neighbouring turbulent air, as demonstrated in Figs. 7 and 8.

The Schlieren structures develop in a timeframe when no high voltage field is present, hence no energy to contribute towards localised heating and subsequent change of density. Further, multiple filaments bridge the gap between the electrode and ground plane with no measurable flow of charge. It is hypothesised that, when the high voltage impulse is applied, heavier local ions (both positive and negative) are attracted towards the charged filaments and move slowly towards them. Once the field has diminished, their momentum continues to carry them into the filament. A higher concentration of ions and electrons remaining within the filaments, whether natural or because they have not recombined, may also attract other local ions and electrons into the region and may collide with neutral molecules also moving them towards the filament. Essentially, the concentrated charged regions pull other ions towards them resulting in a temporary region of differing density around where the charged filaments had existed. The result is a relatively longer-term Schlieren imprint of the short-lived streamers. After around 100 ms, this process is overcome by thermal diffusion with the perpendicular expansion of the filaments being largely consistent with an exponential decay until they disappear into the surrounding air, as shown in Fig. 6. This is consistent with any such gas density differential reaching an equilibrium. However, the more concentrated regions near the electrode and ground plane move towards each other at a much faster rate suggesting an additional mechanism, as also demonstrated in Fig. 6. This is thought to be due to space charge effects, with the ions and electrons gathered at opposing sides of the electrode and ground plane configuration having sufficient charge to pull the regions towards each. Like the filaments, these are also ultimately overcome by thermal diffusion before disappearing into the surrounding environment. It is noted that this mechanism is like, but not the same as, a coronal/electric wind^19^ which is observed during the application of a constant voltage.

**Figure 9:**
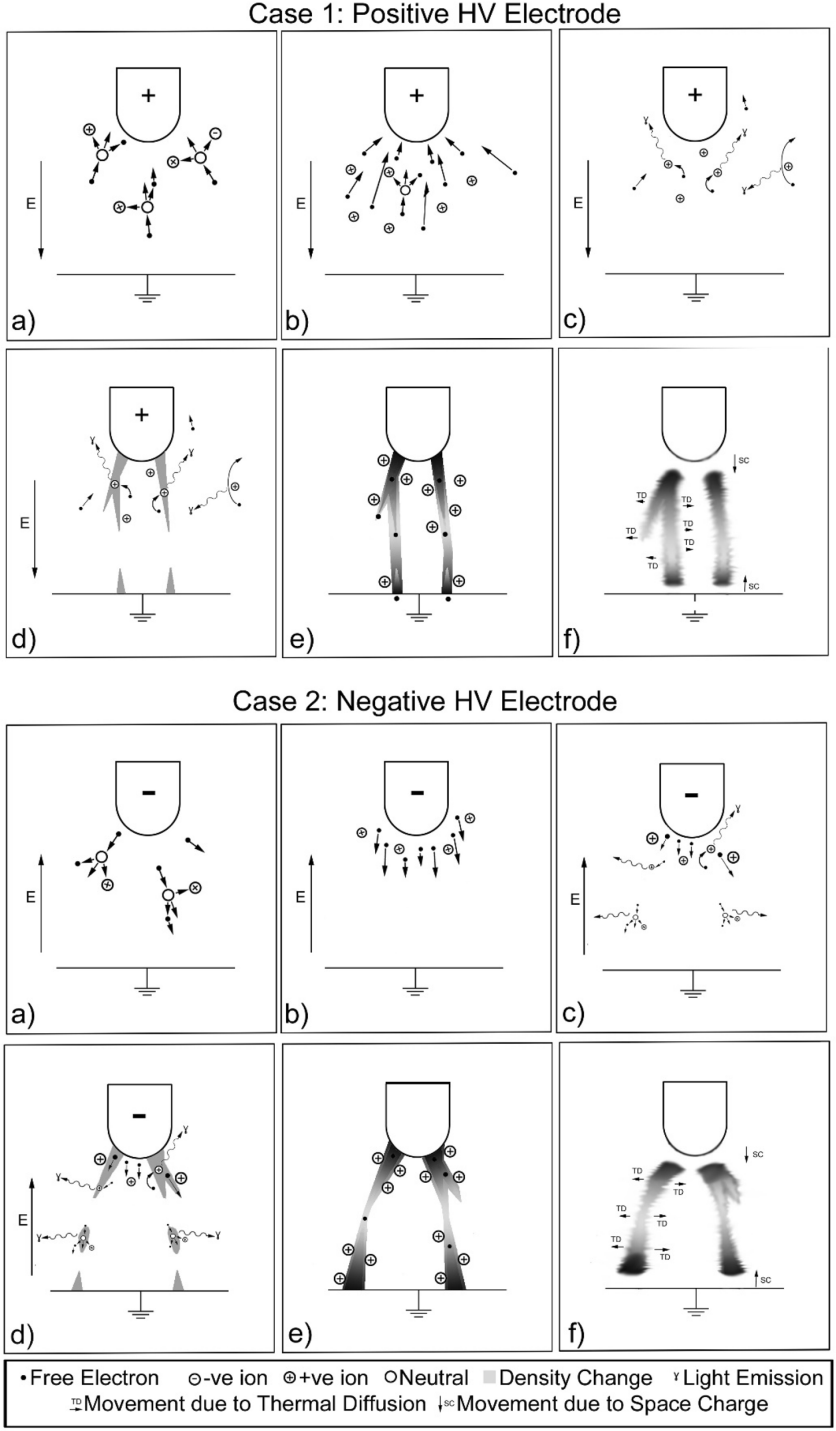
Proposed mechanism for the formation of positive and negative streamers between a pulsed high voltage electrode and ground plane across an air gap. (**a**) Free electrons accelerated towards/away from the electrode creating ions, (**b**) electrons accelerate and collide with more molecules creating an avalanche, (**c**) recombination of ions and Bremsstrahlung radiation emitting light along forming filament structures, (**d**) increase in density around electrode, ground plane and, in the case of negative fields, also in the space between, (**e**) once the field has diminished, the density change is maintained along the filaments creating a Schlieren imprint and (**f**) opposing regions of space charge move towards each other while the Schlieren imprint then diffuses into the surrounding air.

The description given in the introduction of streamer formation between, for example, an energised electrode and a ground plane separated by a small air gap during the application of a high voltage impulse as illustrated in Fig. 1, can hence be modified to the following, as illustrated in Fig. 9. An applied field ionises the air creating an avalanche of accelerated free electrons which emit light, either by recombination or Bremsstrahlung radiation. For positive streamers, filament structures extend out from the electrode whereas, for negative streamers, distinct regions are formed within the air gap. In both cases, several filaments which connect the electrode and ground plane are formed as the ionisation process intensifies. The charged filaments attract local ions towards them which, even after the high voltage field has diminished and the ions have recombined, continue to move into the filaments in the millisecond timeframe creating a Schlieren imprint, whilst regions of opposing space charge at the energised electrode and ground plane start to move towards each other. The imprint and space charge regions are eventually overcome by thermal diffusion as they equalise with the surrounding air. However, during the application of the high voltage field, one of the filaments may reach a critical electron density whilst the high voltage impulse field is applied causing rapid heating, decreasing filament density, and increasing conductivity resulting in an electrical breakdown, with the Schlieren imprint remaining until it dissipates or is disturbed by the turbulent air from the neighbouring breakdown.

In conclusion, the results have demonstrated that light emissions and Schlieren structures coincide along the same streamer filaments, with Schlieren more prominent around the electrode and ground plane where the field intensity is higher, but on different timescales. The high voltage impulse peaked at 50 μs and was fully dissipated by 300 μs, whereas light was emitted only within approximately 100 μs. The Schlieren continued to develop into the microsecond timeframe, moving towards the centre of the air gap whilst diffusing into the surrounding air and fully dissipating within approximately 100 ms. When an electrical breakdown did occur, the Schlieren structures outside the arc remained visible. Such knowledge is important for the understanding and improved design of the insulation strength of insulation gaps subject to high voltage lightning impulse, particularly using the longer-lived Schlieren imprint to understand streamer formation and, subsequently, where a failure or electrical breakdown of the insulation may occur. For example, this is important for the design and manufacture of gas insulated switchgear to better understand partial and full discharge events which can lead to rapid insulation failure result in widespread power distribution failures.

## Method

### Experimental setup

**Figure 10:**
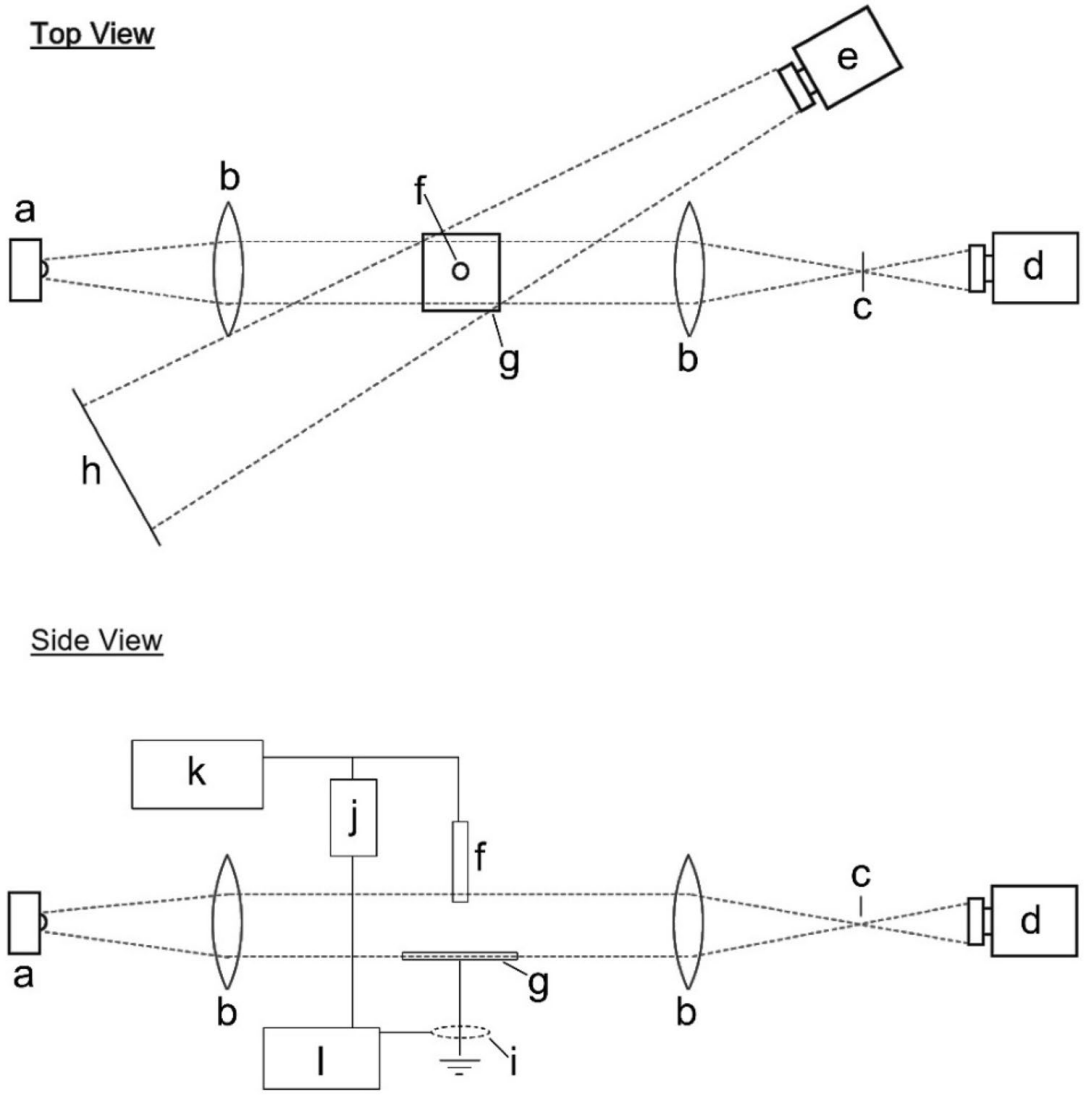
An illustration of the experimental setup. The Schlieren system consists of the (**a**) bright LED light source, (**b**) two biconvex lenses, (**c**) knife edge, (**d**) Schlieren camera, (**e**) visible light camera and (**h**) a flat plane background. The high voltage system consists of the (**f**) electrode, (**g**) ground plane, (**i**) current transducer, (**j**) voltage divider, (**k**) high voltage generator, and (**l**) oscilloscope for reading current and voltage.

The experimental setup is illustrated in Fig. 10 and consisted of two major components: a high voltage system and a camera system. The high voltage system included a commercially available high voltage generator with a resolution of 0.4 kV, connected directly to one of three high voltage electrodes. Each electrode was manufactured from a 1 cm diameter aluminium rod machined to produce a round, flat or pointed end. The oscilloscopes used to record the voltage and current waveforms were triggered from the direct measurement of the voltage rise. The camera system consisted of a pair of synchronised commercially available high-speed cameras and lenses positioned such that they had a clear view of the electrode setup. Both were triggered shortly beforehand in a rolling image format with internal software used to detect a change in the images to capture the event. The Schlieren camera was used in conjunction with an optical setup whereby lenses produced parallel light and a knife edge introduced shading to enhance the Schlieren effect. The light camera was offset at an angle of 20° such that it had a clear view of the electrode arrangement without obstruction from the Schlieren imaging setup. Later, when carrying out more detailed Schlieren type studies, only the Schlieren camera was used, and its settings were adjusted to give a more magnified view with an extended run time. All experiments were conducted in a large electrically isolated room and were remotely controlled.

### Image analysis

**Figure 11:**
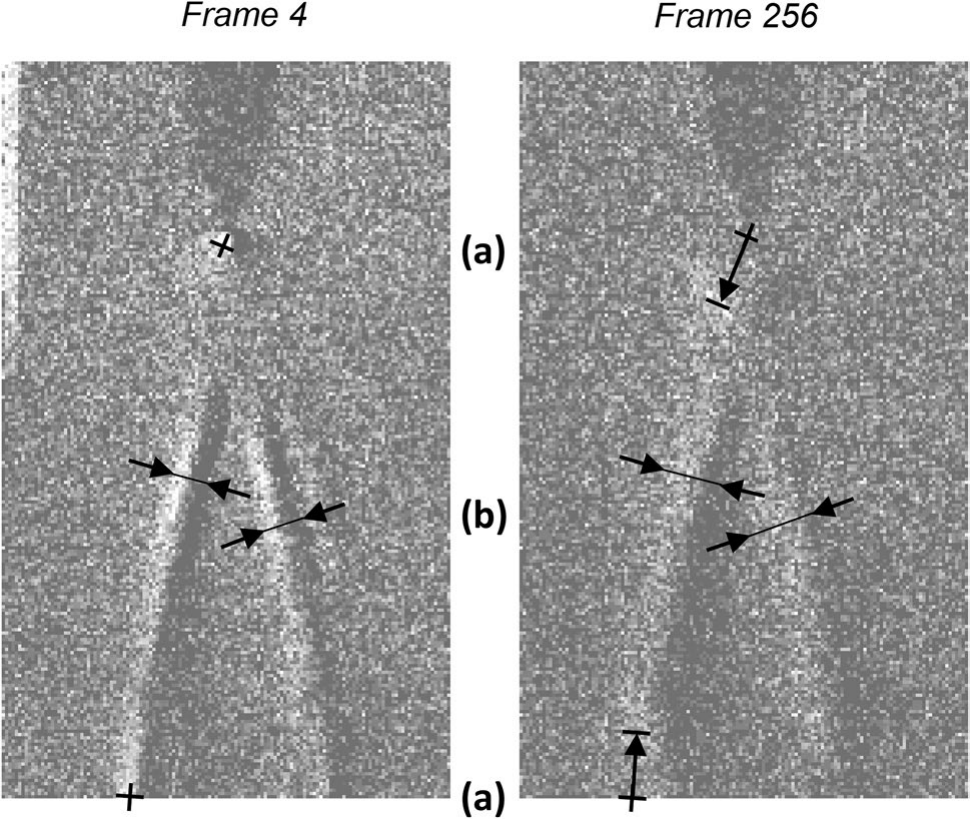
An illustration of (**a**) the measurement of the movement of brighter regions near the electrode and ground plane towards the middle of the air gap and (**b**) the perpendicular expansion of the filaments.

In all cases and for both cameras, the image taken immediately before a streamer image was captured and used as a dark image. The dark image was subtracted from all following images to enhance detail which may not have otherwise been easily visible whilst removing static artefacts present in both images, which were largely found to be a result of the bright LED light source. This technique is the same as given in^13^. The brightness of the images was then increased by 40%, where needed, to emphasise detail for print and display. The perpendicular expansion was measured across pixels which were greater than the background level, whereas the movement of brighter regions was measured by estimating the centre of the region and how far it had moved from its initial position. These steps are demonstrated in Fig. 11.
